# Moderating effect of temperaments between borderline personality traits and mood instability among a sample of Lebanese adults

**DOI:** 10.1371/journal.pone.0343047

**Published:** 2026-02-20

**Authors:** Emmanuelle Awad, Diana Malaeb, Fouad Sakr, Mariam Dabbous, Souheil Hallit, Feten Fekih-Romdhane, Sahar Obeid

**Affiliations:** 1 School of Arts and Sciences, Social and Education Sciences Department, Lebanese American University, Beirut, Lebanon; 2 College of Pharmacy, Gulf Medical University, Ajman, United Arab Emirates; 3 School of Pharmacy, Lebanese International University, Beirut, Lebanon; 4 School of Medicine and Medical Sciences, Holy Spirit University of Kaslik, Jounieh, Lebanon; 5 Applied Science Research Center, Applied Science Private University, Amman, Jordan; 6 The Tunisian Center of Early Intervention in Psychosis, Department of Psychiatry “Ibn Omrane”, Razi hospital (Hopital Razi), Manouba, Tunisia; 7 Tunis El Manar University, Faculty of Medicine of Tunis, Tunis, Tunisia; 8 Department of Psychology and Education, School of Arts and Sciences, Lebanese American University, Jbeil, Lebanon; Federico II University of Naples, ITALY

## Abstract

**Objective:**

The aim of this study is to explore the moderating role of different affective temperaments in the relationship between Borderline Personality traits (BPT) and Mood Instability (MI) among a sample of Lebanese adults.

**Methods:**

This cross-sectional study was conducted in Lebanon in May 2025. An online survey that included the Mood Instability Questionnaire – Trait Short Form (MIQ-T-SF), Temperament Evaluation of Memphis, Pisa, Paris, and San Diego – Modified (TEMPS-M), and McLean Screening Questionnaire for Borderline Personality Disorder (MSI-BPD) was used to collect data.

**Results:**

A total of 872 participants completed the survey, with 66.9% being females and a mean age of 26.97 years. The interactions between borderline personality traits and depressive/cyclothymic/hyperthymic/anxious temperaments were associated with increased mood instability. Higher borderline personality scores were associated with lower (Beta = −0.50; p < 0.001) and higher (Beta = 0.39; p = 0.016) mood instability scores at low and high levels of depressive temperament respectively. Higher borderline personality scores were associated with lower mood instability scores (Beta = −0.51; p < 0.001) at low levels of cyclothymic temperament respectively. At moderate (Beta = 0.67; p < 0.001) and high (Beta = 1.41; p < 0.001) levels of hyperthymic temperament, higher borderline personality scores were associated with higher mood instability scores. Higher borderline personality scores were associated with lower (Beta = −0.37; p = 0.008) and higher (Beta = 0.40; p = 0.012) mood instability scores at low and high levels of anxious temperament respectively. The irritable temperament did not moderate the relationship between borderline personality traits and mood instability.

**Conclusion:**

This study emphasizes the nature of the relationship between affective temperaments, BPT, and MI. These findings are especially important for the Lebanese population, threatened by a rising psychopathology prevalence.

## Introduction

Mood instability (MI) is among maladaptive psychological symptoms that can disrupt functioning, cause distress, and increase impairment. defined by recurrent, persistent, and unpredictable shifts in mood, it is considered a strong predictor of psychological disorders [1]. Adding to that, MI was found to have significant genetic correlations with different psychological disorders [2]. In fact, 12.1% of individuals seeking help due to psychopathology exhibit MI [3]. It was also common among those who were hospitalized for mental health problems, and diagnosed with personality disorders [3]. Notably, MI is a core feature of Borderline Personality traits (BPT) [4].

BPT is characterized by sudden changes in mood, identity, and social relationships [5]. Individuals with BPT display irritability, anger, and fear of abandonment [5]. These explosive symptoms are notorious for making healthcare professionals apprehensive of dealing with individuals diagnosed with or displaying BPT [6], contributing to a mental health crisis. As a result, clinicians are at a higher risk of not recognizing psychological factors associated with BPT [6]. Previously, it was established that BP patients showed high levels of MI in comparison with those who do not have BPT [7]. MI was previously found to be one of the most distinguishing features of BPT among clinical and non-clinical samples [8]. This was also confirmed by a systematic review showing a conclusive relationship between BPT and MI [9]. Having said that, the processes of MI in BPT are still unknown and underexplored in the literature [10]. Moreover, individuals with BPT do not display the same levels of MI amongst themselves [11], suggesting that extraneous factors might manipulate the intensity of shifts in affect. It is imperative to investigate elements that could commonly be linked with this irregularity such as temperaments.

Akiskal, Brieger [12] defined a model that consisted of five distinct temperaments. These five temperament types were hypothesized to be closely related to different MI [12]. The depressive temperament is characterized by persistent sad affect, feelings of guilt, and poor self-image. Meanwhile, the cyclothymic temperament is expressed through unpredictable mood, often fluctuating from positive to low mood. The hyperthymic temperament exhibits feelings of high self-esteem, euphoria, and restlessness. The irritable temperament shows strong irritability, anger, and interpersonal conflict due to strong responses. As for the anxious temperament, it portrays irrational chronic fear and worry [12]. According to Akiskal, Brieger [12], these temperaments are possibly caused by biological predisposition, first appear at the beginning stages of human development, and tend to be stable across the life span.

The links between BPT, MI, and temperaments exist, but the nature and direction of these links remain unclear. To begin with, the five temperaments are trait-like tendencies that might underlie personality mechanisms [13] such those involved in BPT. As for MI, tools such as the Mood Instability Questionnaire (MIQ) include items used from the TEMPS model by Akiskal, Brieger [12], demonstrating the connection between MI and temperaments. TEMPS model is often perceived as a measure to assess MI [14]. Also, affective temperaments were able to predict MI severity, especially in psychological disorders [15]. Abnormal temperamental traits also co-existed with BPT [16]. Another study showed that temperament was a discriminant for BPT in comparison with other psychological disorders [17].

Within the Lebanese context, affective temperaments were positively correlated with personality disorders [18], such as BPT. Adding to that, further results suggest that maladaptive personality variables, BPT included, have temperamental bases [18]. The Lebanese population has endured a multitude of traumatic events and chronic stressors for the past 5 years including a major explosion, economic collapse, and war [19]. These events have caused an increase in personality pathologies and mood lability [20], expected to further heighten in the future [21]. Also, traumatic events and cultural factors were shown to alter mood stability in Lebanon [22]. Adding to that, temperaments, BPT and MI are underexplored in the Lebanese context, which means we lack understanding on the culturally-specific pathways of affective instability. This is especially relevant given that temperaments differ from culture to culture, favoring ones that help individuals accommodate to their environment, which was previously observed in the Lebanese population [23]. Therefore, the aim of this study is to explore the moderating role of different temperaments in the relationship between BPT and MI. We can define which temperaments may be associated with the relationship between BPT and MI.

## Methods

### Study design

This study employed a cross-sectional design. Data were collected using a 20-minute survey administered via Google Forms and distributed through social media platforms. A convenience and snowball sampling method was used to recruit 872 participants. In this method, initial participants were invited to complete the survey and encouraged to share it with their contacts, allowing the sample to grow through referrals. All Lebanese residents and citizens who were adults and had Internet access were eligible to participate. Informed consent was obtained from all participants before they competed the survey, and their responses remained anonymous. Data collection took place between May 7 and May 31, 2025.

### Minimal sample size calculation

A minimal sample of 411 was deemed necessary using the formula suggested by Fritz and MacKinnon [24] to estimate the sample size: $n=\frac{L}{f\text{2}}+k+\text{1}$, where f = 0.14 for small effect size, L = 7.85 for an α error of 5% and power β = 80%, and k = 9 variables to be entered in the model.

### Questionnaire

We ensured full anonymity and confidentiality throughout the study. The questionnaire included inquiries regarding sociodemographic factors such as age, gender, education level, marital status, living situation, and Household Crowding Index (HCI). The HCI is determined by dividing the total household population (excluding newborns) by the number of rooms in the residence, excluding the kitchen [25]. The strength, frequency, and duration of physical activity were multiplied to create the physical activity index, with scores varying from 1 to 100 [26].

#### Mood Instability Questionnaire – Trait Short Form (MIQ-T-SF).

The MIQ-T-SF is a 17-item scale designed to measure MI. Statements are scored from “0=Not at all true” to “5=Exactly true). Examples of the statements include: “I constantly switch from being lively and sluggish.” and “My mood changes depending on the season.” Higher scores indicate higher MI. The scale exhibited high internal consistency in the current study (α = 0.91) [27].

#### Temperament Evaluation of Memphis, Pisa, Paris, and San Diego – Modified (TEMPS-M).

The TEMPS-M is a scale meant to measure the five affective temperaments: depressive, cyclothymic, hyperthymic, irritable and anxious. It includes 35 items, with 7 items measuring each temperament. Each item is scored from “0=Not at all” to “5=Extremely”. Higher scores indicated stronger intensity on temperament. The subscales demonstrated good internal consistency in the current study (depressive: α = 0.77, cyclothymic: α = 0.87, hyperthymic: α = 0.90, irritable: α = 0.86, and anxious: α = 0.85). The scale is validated in Arabic for the Lebanese population [18].

#### McLean Screening Questionnaire for Borderline Personality Disorder (MSI-BPD).

The MSI-BPD is used to measure BPT. The scale includes 10 questions, answered by “yes” or “no”. Each “yes” accounts for 1 point. A sum equal or above 7 points indicates higher probability of BPT. Examples of questions from the scale include: “Have you chronically felt empty?” and “Have you been extremely moody?”. The scale showed good internal consistency in the current study (α = 0.79) [28].

### Ethical considerations

Ethics approval for this study was obtained from the ethics committee of the School of Pharmacy at the Lebanese International University (2025ERC-019-LIUSOP). Written informed consent was obtained from all subjects; the online submission of the soft copy was considered equivalent to receiving a written informed consent. All methods were performed in accordance with the relevant guidelines and regulations.

### Statistical analysis

The SPSS v.27 software was used for the statistical analysis. The Student t test was used to compare a continuous variable and a dichotomous variable, and Pearson’s test to correlate two continuous variables. The moderation analysis was performed using PROCESS Macro v.4.2 Model 1. In all moderation models, the independent variable (borderline personality traits) and the moderator (each temperament) were mean-centered prior to the computation of the interaction term, in accordance with the default settings of the PROCESS Macro. All covariates were entered identically across all moderation models. Although the MSI is commonly used as a screening tool, it was analyzed as a continuous measure to capture dimensional variability in borderline personality traits. Interaction terms were probed by examining the association of borderline personality traits with mood instability at −1 SD, mean and +1 SD of the moderator (each temperament). Covariates entered in the model were those that showed a p < 0.25 in the bivariate analysis. P < 0.05 was considered statistically significant.

## Results

The MIQ score was normally distributed as shown by its skewness and kurtosis values between ±1. In total, 872 participants completed the questionnaire, with 66.9% females and a mean age of 26.97 years. The full description of the sample is in Table 1.

**Table 1:** Sociodemographic and other characteristics of the sample (N = 872).

Variable	N (%)
**Gender**	
Male	289 (33.1%)
Female	583 (66.9%)
**Marital status**	
Single, divorced or widowed	641 (73.5%)
Married	231 (26.5%)
**Living situation**	
Alone	81 (9.3%)
With family	771 (88.4%)
With friends	20 (2.3%)
**Education**	
School level	138 (15.8%)
University level	734 (84.2%)
	**Mean ± SD**
Age (years)	26.97 ± 10.29
Household crowding index (person/room)	1.16 ± 0.60
Physical activity	26.33 ± 20.35
Mood instability	14.61 ± 9.87
Borderline personality	3.60 ± 2.77
Depressive temperament	16.23 ± 5.79
Cyclothymic temperament	17.25 ± 5.76
Hyperthymic temperament	19.97 ± 6.22
Irritable temperament	15.94 ± 5.66
Anxious temperament	17.40 ± 6.03

The borderline personality traits score was normally distributed (skewness = 0.339 and kurtosis = −0.617), with 175 (18.7%) of participants having a score of 0 whereas 39 (4.2%) scored 10. Moreover, 126 (13.5%) screened positive for probable borderline personality disorder (score of ≥7).

### Bivariate analysis of factors associated with mood instability

A higher mean MIQ score was found in females vs males, in single participants vs married and in those with a university level of education vs secondary or less (Table 2). Furthermore, higher MIQ scores were associated with higher levels of borderline personality traits, and with all temperament subscales, whereas older age was associated with lower MIQ scores (Table 3).

**Table 2:** Bivariate analysis of factors associated with mood instability.

Variable	Mean ± SD	t	Df	*P*	Effect size
**Gender**		−3.07	870	**0.002**	0.221
Male	13.16 ± 9.20				
Female	15.33 ± 10.11				
**Marital status**		4.80	870	**<0.001**	0.368
Single, divorced or widowed	15.56 ± 10.03				
Married	11.97 ± 8.92				
**Living situation**		1.71	2, 869	0.182	0.004
Alone	15.01 ± 8.57				
With family	14.47 ± 9.96				
With friends	18.50 ± 10.79				
**Education level**		−2.61	870	**0.009**	0.242
Secondary or less	12.61 ± 9.22				
University	14.99 ± 9.95				

Bold numbers indicate significant p value.

**Table 3:** Pearson correlation matrix.

	1	2	3	4	5	6	7	8	9
1. Mood instability	1								
2. Borderline personality	0.13***	1							
3. Depressive temperament	0.54***	0.29***	1						
4. Cyclothymic temperament	0.66***	0.28***	0.76***	1					
5. Hyperthymic temperament	0.49***	−0.06	0.28***	0.38***	1				
6. Irritable temperament	0.46***	0.29***	0.73***	0.63***	0.31***	1			
7. Anxious temperament	0.57***	0.25***	0.68***	0.67***	0.35***	0.61***	1		
8. Age	−0.14***	−0.07*	−0.11**	−0.16***	−0.14***	−0.10**	−0.11**	1	
9. HCI	0.03	0.02	−0.003	0.03	−0.03	−0.03	0.03	−0.03	1
10. PAI	−0.05	0.04	0.01	−0.03	0.03	0.05	−0.06	−0.05	−0.10**

HCI = Household crowding index; PAI = Physical activity index. **p < 0.01; ***p < 0.001.

### Analysis of moderation

All moderation models were adjusted for gender, marital status, education level, living situation, age and physical activity. Because both borderline personality traits and temperament dimensions were mean-centered, the main effect of borderline personality traits represents its association with mood instability at the mean level of the temperament dimension, while the interaction term indicates whether this association varies across levels of temperament.

**Fig 1:**
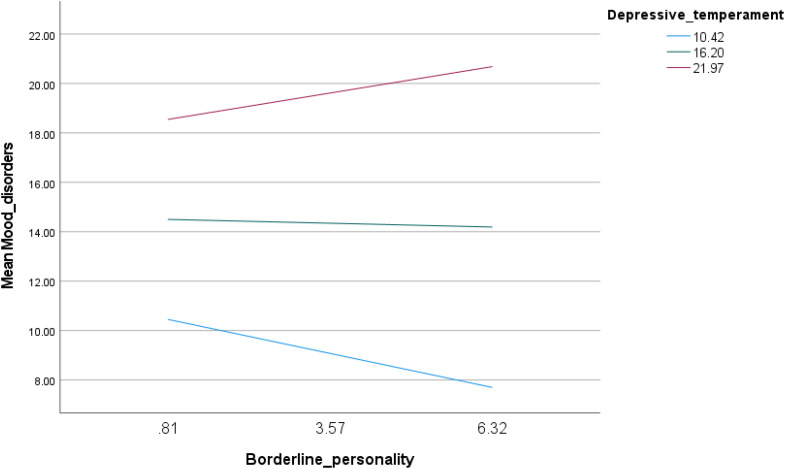
Interaction of borderline personality by depressive temperament with mood instability.

The interaction between borderline personality traits and depressive temperament was associated with increased mood instability (Beta = 0.08; p < 0.001) (Table 4, Model 1). Examination of conditional effects revealed a cross-over interaction pattern: higher borderline personality scores were associated with lower mood instability at low levels of depressive temperament (Beta = −0.50; p < 0.001), whereas they were associated with higher mood instability at high levels of depressive temperament (Beta = 0.39; p = 0.016) (Table 5, Model 1 and Fig 1).

**Table 4:** Moderation analysis taking borderline personality as the independent variable, each temperament as the moderator and mood instability as the dependent variable.

	Beta	*T*	*P*	95% CI
**Model 1: Depressive temperament as the moderator**				
Borderline personality	−1.30	−4.35	**<0.001**	−1.89; −0.71
Depressive temperament	0.64	7.64	**<0.001**	0.47; 0.80
Interaction borderline personality by depressive temperament	0.08	4.26	**<0.001**	0.04; 0.11
**Model 2: Cyclothymic temperament as the moderator**				
Borderline personality	−1.24	−4.55	**<0.001**	− 1.77; −0.70
Cyclothymic temperament	0.88	11.62	**<0.001**	0.73; 1.03
Interaction borderline personality by cyclothymic temperament	0.06	4.06	**<0.001**	0.03; 0.09
**Model 3: Hyperthymic temperament as the moderator**				
Borderline personality	−1.70	−5.25	**<0.001**	− 2.34; −1.07
Hyperthymic temperament	0.35	4.86	**<0.001**	0.21; 0.50
Interaction borderline personality by hyperthymic temperament	0.12	7.32	**<0.001**	0.09; 0.15
**Model 4: Irritable temperament as the moderator**				
Borderline personality	−0.58	−1.79	0.074	− 1.22; 0.06
Irritable temperament	0.67	7.47	**<0.001**	0.49; 0.85
Interaction borderline personality by irritable temperament	0.04	1.86	0.063	−0.002; 0.08
**Model 5: Anxious temperament as the moderator**				
Borderline personality	−1.09	−3.61	**<0.001**	−1.68; −0.50
Anxious temperament	0.67	8.40	**<0.001**	0.51; 0.82
Interaction borderline personality by anxious temperament	0.06	3.73	**<0.001**	0.03; 0.10

Numbers in bold indicate significant p values.

**Table 5:** Conditional effect of the focal predictor (borderline personality) at values of the moderator (each temperament).

	Beta	*t*	*P*	95% CI
**Model 1: Depressive temperament as the moderator**				
Low (= 10.42)	−0.50	−3.52	**<0.001**	−0.78; −0.22
Moderate (= 16.20)	−0.06	−0.51	0.614	−0.27; 0.16
High (= 21.97)	0.39	2.41	**0.016**	0.07; 0.70
**Model 2: Cyclothymic temperament as the moderator**				
Low (= 11.48)	−0.51	−4.11	**<0.001**	−0.75; −0.27
Moderate (= 17.25)	−0.14	−1.42	0.156	−0.34; 0.05
High (= 23.01)	0.22	1.53	0.127	−0.06; 0.51
**Model 3: Hyperthymic temperament as the moderator**				
Low (= 13.77)	−0.07	−0.49	0.622	−0.33; 0.20
Moderate (= 19.99)	0.67	6.28	**<0.001**	0.46; 0.88
High (= 26.20)	1.41	8.89	**<0.001**	1.10; 1.72
**Model 4: Anxious temperament as the moderator**				
Low (= 11.36)	−0.37	−2.65	**0.008**	−0.64; −0.10
Moderate (= 17.38)	0.02	0.14	0.890	−0.20; 0.23
High (= 23.41)	0.40	2.51	**0.012**	0.09; 0.71

Numbers in bold indicate significant p values.

**Fig 2:**
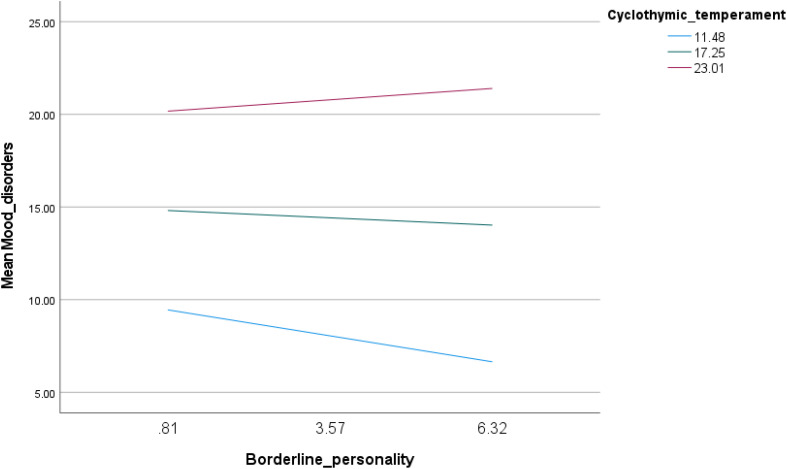
Interaction of borderline personality by cyclothymic temperament with mood instability.

Similarly, the interaction between borderline personality traits and cyclothymic temperament was associated with increased mood instability (Beta = 0.06; p < 0.001) (Table 4, Model 2). Conditional effects showed that borderline personality traits were negatively associated with mood instability at low levels of cyclothymic temperament (Beta = −0.51; p < 0.001) (Table 5, Model 2 and Fig 2).

**Fig 3:**
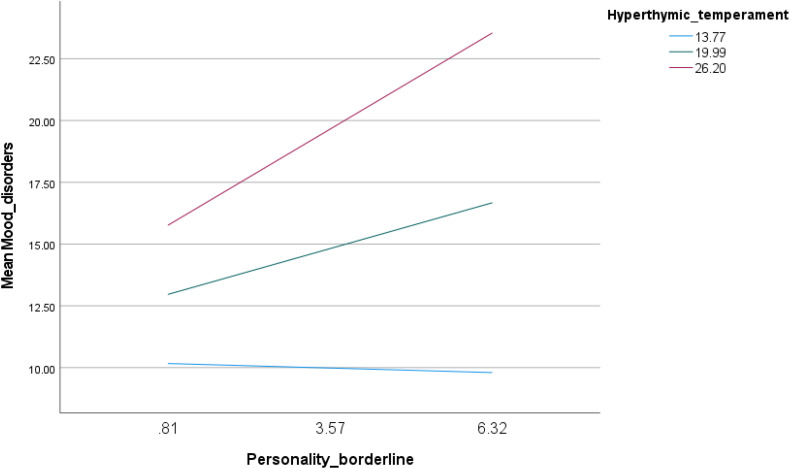
Interaction of borderline personality by hyperthymic temperament with mood instability.

For hyperthymic temperament, the interaction between borderline personality traits and hyperthymic temperament was associated with increased mood instability (Beta = 0.12; p < 0.001) (Table 4, Model 3). Conditional effects demonstrated a strengthening interaction: borderline personality traits were significantly associated with higher mood instability at moderate (Beta = 0.67; p < 0.001) and high (Beta = 1.41; p < 0.001) levels of hyperthymic temperament (Table 5, Model 3 and Fig 3).

The irritable temperament did not moderate the relationship between borderline personality traits and mood instability (Table 4, Model 4).

**Fig 4:**
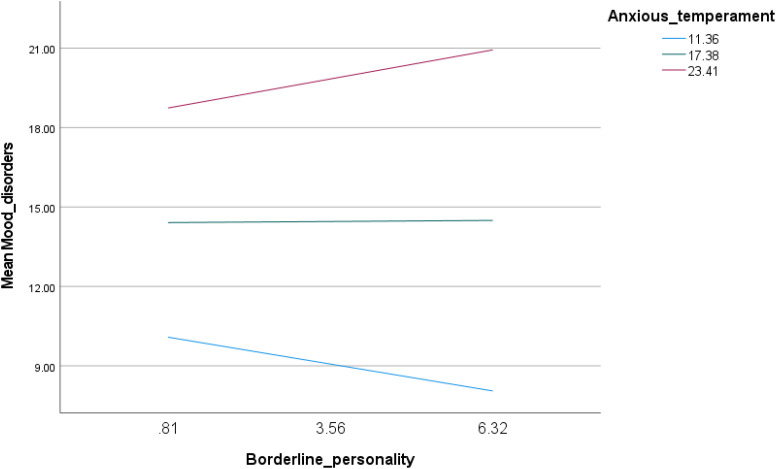
Interaction of borderline personality by anxious temperament with mood instability.

The interaction between borderline personality traits and anxious temperament was associated with increased mood instability (Beta = 0.06; p < 0.001) (Table 4, Model 5). A cross-over interaction was observed, such that more pronounced traits of borderline personality were associated with lower mood instability at low levels of anxious temperament (Beta = −0.37; p = 0.008) and with higher mood instability at high levels of anxious temperament (Beta = 0.40; p = 0.012) (Table 5, Model 4 and Fig 4).

## Discussion

The results of our moderation analysis showed that the depressive temperament moderated the relationship between BPT and MI. Higher BPT was associated with lower and higher MI at low and high levels of depressive temperament. This could indicate that BPT is only associated with MI when depressive temperament is high. Individuals who have a depressive temperament tend to be chronically pessimistic and are more vulnerable to sadness and helplessness [13]. Coupled with BPT, they might experience pervasive distress and could lack good management when dealing with intense changes in identity, affect, and social relationships [29]. Alternatively, low depressive temperament might reduce the probability of dealing poorly with BPT, possibly mitigating MI. Research suggests that individuals with BPT show a different depressive quality, in comparison with those who do not have BPT [30]. In another study, it was suggested that different BPT subtypes exist based on temperaments [31]. These results could propose a hypothesis: temperaments may be linked to BP outcomes and manifestations, such as MI.

As for the cyclothymic temperament, it moderated the relationship between BPT and MI. Higher BPT was associated with lower MI at low levels of cyclothymic temperament. This indicates that if an individual exhibits high BPT but has low cyclothymic temperament, they will show low MI. This result suggests that high BPT and high MI are not exclusive, further enforcing that BPT patients may show MI that is different in severity [11], potentially associated by their type of temperament. The cyclothymic temperament is most characterized by frequent and intense mood swings [29]. In this case, given that the cyclothymic temperament is low, the possible enforcer of MI is at a low level. For that reason, high BP could be associated with low MI when moderated by low cyclothymic temperament.

At the same time, the hyperthymic temperament moderated the relationship between borderline personality and mood instability. At moderate and high levels of hyperthymic temperament, higher borderline personality was associated with higher mood instability. Although the hyperthymic temperament consists of tendencies to be optimistic, more resistant to negative events, and exhibiting positive mood, it can also feature uninhibited behavior [13]. When present with BPT, it can translate into higher impulsivity and lower deliberation of emotion, thought, and behavior. We can hypothesize that these interactions can present higher MI in this case.

Finally, the anxious temperament moderated the relationship between borderline personality and mood instability. Higher borderline personality was associated with lower and higher mood instability at low and high levels of anxious temperament. These findings imply that if someone does not have an anxious disposition, they are less likely to exhibit MI when they have BPT. Meanwhile, if a person has a high anxious temperament, they would be more likely to show severe MI if they have BPT. In a previous study, the anxious temperament was associated with chronic fear of people abandonment [29]. Fear of abandonment is also a central element of BPT [32]. It can be hypothesized that having anxiety and anticipating abandonment, coupled with fear of abandonment due to BPT, can exacerbate agitation and subsequently MI.

Women who are single and have a university level education showed higher MI. A meta-analysis revealed that men of older age showed higher comorbid BP and MI. Having said that, a recent review concluded that the literature lacks studies that investigate the BP prevalence differences among men and women [33]. Yet, men and women who met the criteria for BP were differentiated by distinct temperaments [33]. For example, women from a clinical sample had a higher probability of scoring high on the cyclothymic temperament instead of the hyperthymic temperament in a previous study [34].

Although no research exists about women’s relationship status and MI, single women report poorer mental health than those married [35]. This can be explained by results showing that married women had higher perceived social support, mitigating poor mental health outcomes [36]. Paradoxically, women who have a university education might have more mental health awareness, potentially increasing the possibility of reporting MI [37]. In our culturally-specific context, single women who are more educated might encounter higher societal pressure to get married or achieve highly in their career [38]. As a result, they might suffer from poor mental health outcomes such as MI [38].

Meanwhile, older age was associated with lower MI. This finding is consistent with previous ones as evidence suggests that older adults are better at emotion regulation [39], which could potentially lower MI.

Also, individuals with higher scores on BPT and all temperament subscales showed higher MI. As previously mentioned, temperaments are trait-like qualities similar to personality elements [40,41]. BPT is also a personality-related pathology [42]. Both build an environment that fosters MI [9,29]. As a result, MI might be higher in participants who scored higher on BPT and all temperaments.

### Limitations

Some limitations need to be noted. First, the MIQ-T-SF and MSI-BPD is not validated for the Lebanese population. While internal consistency in the present study was acceptable, future studies should confirm their factorial structure and measurement invariance in Lebanon. Moreover, there is potential conceptual and content overlap between the MIQ-T-SF and the TEMPS-M, as both instruments originate from the affective temperament framework and are commonly used to assess aspects of mood instability. This raises the possibility that part of the observed moderation effects may be influenced by shared variance rather than entirely distinct constructs. The MIQ-T-SF primarily assesses mood instability as a dynamic outcome, whereas the TEMPS-M captures relatively stable affective temperaments conceptualized as enduring traits. Although the MIQ-T-SF and TEMPS-M originate from the same affective framework and are related, mood instability and affective temperaments are distinct constructs. Mood instability represents fast changes in affective states and changes in affective states over time [4]. Meanwhile, affective temperaments are stable, relatively temporally consistent, and are trait-like affective states [13]. Second, the data was collected using self-report measures, increasing the possibility of responder’s bias. Also, the snowball sampling techniques does not allow generalizability of the presented results in the Lebanese population. It is also important to highlight that the study investigated traits and temperaments exclusively using self-report measures, rather than structured clinical interviews and formal diagnosis of psychopathology, which can contribute to theoretical clarification. Future research can reproduce these associations by using representative sampling in clinical populations. Finally, given the cross-sectional design, we cannot establish the directionality of effects between temperaments, BPT, and MI. Our findings should be interpreted as associations rather than causal relationships.

### Clinical implications

To begin with, our results raise the importance of screening for MI in individuals who might not exhibit pathology and show adaptive behavior such as younger educated women. Second, it could help clinicians identify BPT and MI high-risk populations based on assessing affective temperaments. The early identification of maladaptive affective temperaments can potentially highlight associated psychological symptoms such as BPT and MI. This can be done by tailoring emotion regulation strategies and therapeutic techniques for specific temperaments, enhancing treatment personalization. The current results can aid care providers and mental health professionals in vulnerable settings such as Lebanon in providing assistance for individuals susceptible to psychopathology.

## Conclusion

The moderation role of affective temperaments between BPT and MI is highlighted by the current results, specifically the depressive, cyclothymic, hyperthymic, and anxious temperaments. These findings are especially important for the Lebanese population, threatened by a rising psychopathology prevalence. This study emphasizes the nature of the relationship between affective temperaments, BPT, and MI. Thus, allowing mental health professionals to be better equipped when dealing with individuals exhibiting BPT and MI.

## References

[1] Fristad MA. Mood instability: what it is, why it matters, and what to do about it. Elsevier; 2022. p. 1224–6. 10.1016/j.jaac.2022.03.012 35346786

[2] Hindley G, O’Connell KS, Rahman Z, Frei O, Bahrami S, Shadrin A, et al. The shared genetic basis of mood instability and psychiatric disorders: a cross-trait genome-wide association analysis. Am J Med Genet B Neuropsychiatr Genet. 2022;189(6):207–18. doi: 10.1002/ajmg.b.32907 35841185 PMC9541703

[3] Patel R, Lloyd T, Jackson R, Ball M, Shetty H, Broadbent M, et al. Mood instability is a common feature of mental health disorders and is associated with poor clinical outcomes. BMJ Open. 2015;5(5):e007504. doi: 10.1136/bmjopen-2014-007504 25998036 PMC4452754

[4] Broome MR, Saunders KEA, Harrison PJ, Marwaha S. Mood instability: significance, definition and measurement. Br J Psychiatry. 2015;207(4):283–5. doi: 10.1192/bjp.bp.114.158543 26429679 PMC4589661

[5] Leichsenring F, Heim N, Leweke F, Spitzer C, Steinert C, Kernberg OF. Borderline personality disorder: a review. JAMA. 2023;329(8):670–9. doi: 10.1001/jama.2023.0589 36853245

[6] Gunderson JG, Herpertz SC, Skodol AE, Torgersen S, Zanarini MC. Borderline personality disorder. Nat Rev Dis Primers. 2018;4:18029. doi: 10.1038/nrdp.2018.29 29795363

[7] Abolalaei A, Atadokht A, Basharpoor S. Comparison of impulsivity, emotional instability, decision making and risky behaviors in patients with bipolar disorder and borderline personality disorder. J Res Psychopathol. 2022;3(10):1–11.

[8] Carvalho L de F, Pianowski G. Dependency, mood instability, and inconsequence traits for discriminating borderline personality disorder. Trends Psychiatry Psychother. 2019;41(1):78–82. doi: 10.1590/2237-6089-2018-0010 30994782

[9] D’Aurizio G, Di Stefano R, Socci V, Rossi A, Barlattani T, Pacitti F, et al. The role of emotional instability in borderline personality disorder: a systematic review. Ann Gen Psychiatry. 2023;22(1):9. doi: 10.1186/s12991-023-00439-0 36918920 PMC10011773

[10] Schmidt P, Tewes C, Stanghellini G. Nobody? Disturbed self-experience in borderline personality disorder and four kinds of instabilities. In: Time and body: phenomenological and psychopathological approaches. 2020. p. 206–29.

[11] Dixon-Gordon KL, Laws H. Emotional variability and inertia in daily life: links to borderline personality and depressive symptoms. J Pers Disord. 2021;35(Suppl A):162–71. doi: 10.1521/pedi_2021_35_504 33650892

[12] Akiskal HS, Brieger P, Mundt C, Angst J, Marneros A. Temperament and affective disorders. The TEMPS-A Scale as a convergence of European and US-American concepts. Nervenarzt. 2002;73(3):262–71. doi: 10.1007/s00115-001-1230-y 11963262

[13] Akiskal HS, Akiskal KK, Haykal RF, Manning JS, Connor PD. TEMPS-A: progress towards validation of a self-rated clinical version of the Temperament Evaluation of the Memphis, Pisa, Paris, and San Diego Autoquestionnaire. J Affect Disord. 2005;85(1–2):3–16. doi: 10.1016/j.jad.2004.12.001 15780671

[14] Yoon J, Ha TH, Oh S, Park YS, Ryoo HA, Yu H-A, et al. Development and validation of the Mood Instability Questionnaire-Trait (MIQ-T). Medicina (Kaunas). 2021;57(8):838. doi: 10.3390/medicina57080838 34441044 PMC8399587

[15] Miola A, Baldessarini RJ, Pinna M, Tondo L. Relationships of affective temperament ratings to diagnosis and morbidity measures in major affective disorders. Eur Psychiatry. 2021;64(1):e74. doi: 10.1192/j.eurpsy.2021.2252 34812134 PMC8715280

[16] Massó Rodriguez A, Hogg B, Gardoki-Souto I, Valiente-Gómez A, Trabsa A, Mosquera D, et al. Clinical features, neuropsychology and neuroimaging in bipolar and borderline personality disorder: a systematic review of cross-diagnostic studies. Front Psychiatry. 2021;12:681876. doi: 10.3389/fpsyt.2021.681876 34177664 PMC8220090

[17] Kouros I, Holmberg H, Ekselius L, Ramklint M. Temperament, but not childhood trauma, distinguishes borderline personality disorder from bipolar disorder and ADHD. Nord J Psychiatry. 2024;78(1):79–86. doi: 10.1080/08039488.2023.2267041 37870069

[18] Fekih-Romdhane F, Yakın E, Bitar Z, Malaeb D, Sawma T, Obeid S, et al. Validation of the Arabic version of the 35-item TEMPS-M in a community sample of adults. BMC Psychol. 2023;11(1):28. doi: 10.1186/s40359-023-01064-y 36709317 PMC9883938

[19] Karam EG, El-Jamal M, Osman R, Toukan S, Mouawad GI, Al Barathie J. The aftermath of multiple trauma on a nation: unraveling Lebanon’s unique mental health struggle. Front Psychiatry. 2025;15:1444245. doi: 10.3389/fpsyt.2024.1444245 39876996 PMC11773410

[20] Khalil MB. La violence de la guerre civile: hôte du trouble de la personnalité limite. InteraXXIons. 2024;4:81–96.

[21] Farran N. Mental health in Lebanon: Tomorrow’s silent epidemic. Ment Health Prev. 2021;24:200218. doi: 10.1016/j.mhp.2021.200218 34660191 PMC8503814

[22] Hasbany D. The influence of age and traumatic experience on emotional awareness in a Lebanese context: a correlational study. 2024.

[23] Gonda X, Vázquez GH, Akiskal KK, Akiskal HS. From putative genes to temperament and culture: cultural characteristics of the distribution of dominant affective temperaments in national studies. J Affect Disord. 2011;131(1–3):45–51. doi: 10.1016/j.jad.2010.12.003 21195481

[24] Fritz MS, Mackinnon DP. Required sample size to detect the mediated effect. Psychol Sci. 2007;18(3):233–9. doi: 10.1111/j.1467-9280.2007.01882.x 17444920 PMC2843527

[25] Melki IS, Beydoun HA, Khogali M, Tamim H, Yunis KA, National Collaborative Perinatal Neonatal Network (NCPNN). Household crowding index: a correlate of socioeconomic status and inter-pregnancy spacing in an urban setting. J Epidemiol Community Health. 2004;58(6):476–80. doi: 10.1136/jech.2003.012690 15143115 PMC1732777

[26] Assaf M, Hallit R, Fawaz M, Malaeb D, El Khatib S, Fekih-Romdhane F, et al. Arabic translation and psychometric testing of the physical activity index (PAI). Lifestyle Med. 2026;7. 10.1002/lim2.70052

[27] Yoon J, Yu H, Jang Y, Lee D, Park YS, Ihm HK, et al. Validation of the short form of the Mood Instability Questionnaire-Trait (MIQ-T-SF) in the Korean general population. Psychiatry Investig. 2023;20(5):408–17. doi: 10.30773/pi.2022.0275 37253466 PMC10232051

[28] Zanarini MC, Vujanovic AA, Parachini EA, Boulanger JL, Frankenburg FR, Hennen J. A screening measure for BPD: the McLean Screening Instrument for Borderline Personality Disorder (MSI-BPD). J Pers Disord. 2003;17(6):568–73. doi: 10.1521/pedi.17.6.568.25355 14744082

[29] Lee H, Eom Y, Lee J, Lee D, Yu H, Kang HS, et al. Interconnectedness of borderline personality pathology and affective temperaments in patients with mood disorders: a network analysis. Nord J Psychiatry. 2025;79(2):110–9. doi: 10.1080/08039488.2025.2451370 39827408

[30] Köhling J, Ehrenthal JC, Levy KN, Schauenburg H, Dinger U. Quality and severity of depression in borderline personality disorder: a systematic review and meta-analysis. Clin Psychol Rev. 2015;37:13–25. doi: 10.1016/j.cpr.2015.02.002 25723972

[31] Sleuwaegen E, Claes L, Luyckx K, Berens A, Vogels C, Sabbe B. Subtypes in borderline patients based on reactive and regulative temperament. Pers Individ Dif. 2017;108:14–9.

[32] Palihawadana V, Broadbear JH, Rao S. Reviewing the clinical significance of “fear of abandonment” in borderline personality disorder. Australas Psychiatry. 2019;27(1):60–3. doi: 10.1177/1039856218810154 30403145

[33] Bozzatello P, Blua C, Brandellero D, Baldassarri L, Brasso C, Rocca P, et al. Gender differences in borderline personality disorder: a narrative review. Front Psychiatry. 2024;15:1320546. doi: 10.3389/fpsyt.2024.1320546 38283847 PMC10811047

[34] Perugi G, Toni C, Maremmani I, Tusini G, Ramacciotti S, Madia A, et al. The influence of affective temperaments and psychopathological traits on the definition of bipolar disorder subtypes: a study on bipolar I Italian national sample. J Affect Disord. 2012;136(1–2):e41–9. doi: 10.1016/j.jad.2009.12.027 20129674

[35] Grundström J, Konttinen H, Berg N, Kiviruusu O. Associations between relationship status and mental well-being in different life phases from young to middle adulthood. SSM Popul Health. 2021;14:100774. doi: 10.1016/j.ssmph.2021.100774 33869721 PMC8040327

[36] Vaingankar JA, Abdin E, Chong SA, Shafie S, Sambasivam R, Zhang YJ, et al. The association of mental disorders with perceived social support, and the role of marital status: results from a national cross-sectional survey. Arch Public Health. 2020;78:108. doi: 10.1186/s13690-020-00476-1 33133595 PMC7592592

[37] Pehlivan Ş, Tokur Kesgin M, Uymaz P. Psychological distress and mental health literacy in university students. Perspect Psychiatr Care. 2021;57(3). 10.1111/ppc.12709 33330978

[38] Bou Malhab S, Sacre H, Malaeb D, Lahoud N, Khachman D, Azzi J, et al. Factors related to autonomy among Lebanese women: a web-based cross-sectional study. BMC Womens Health. 2021;21(1):369. doi: 10.1186/s12905-021-01501-3 34670539 PMC8527961

[39] Allen VC, Windsor TD. Age differences in the use of emotion regulation strategies derived from the process model of emotion regulation: a systematic review. Aging Ment Health. 2019;23(1):1–14. doi: 10.1080/13607863.2017.1396575 29148830

[40] Murillo AS, Clifford S, Cheng CH, Doane LD, Davis MC, Lemery-Chalfant K. Development of temperament types from infancy to adolescence: Genetic and environmental influences with an economically and racially/ethnically diverse sample. Dev Psychol. 2024;60(11):2200–19. doi: 10.1037/dev0001828 39172413 PMC11866458

[41] Muris P, Bakker I, Peulen M, van Mulekom S, Meesters C. The good, the bad, and the ugly: a comprehensive study of temperament and personality traits as correlates of self-reported disruptive behavior problems in male and female adolescents. Front Child Adolesc Psychiatry. 2023;2:1173272. doi: 10.3389/frcha.2023.1173272 39816877 PMC11731598

[42] Leichsenring F, Fonagy P, Heim N, Kernberg OF, Leweke F, Luyten P, et al. Borderline personality disorder: a comprehensive review of diagnosis and clinical presentation, etiology, treatment, and current controversies. World Psychiatry. 2024;23(1):4–25. 38214629 10.1002/wps.21156 PMC10786009

